# Screening of Parkinsonian subtle fine-motor impairment from touchscreen typing via deep learning

**DOI:** 10.1038/s41598-020-69369-1

**Published:** 2020-07-28

**Authors:** Dimitrios Iakovakis, K. Ray Chaudhuri, Lisa Klingelhoefer, Sevasti Bostantjopoulou, Zoe Katsarou, Dhaval Trivedi, Heinz Reichmann, Stelios Hadjidimitriou, Vasileios Charisis, Leontios J. Hadjileontiadis

**Affiliations:** 1 Department of Electrical and Computer Engineering, Aristotle University of Thessaloniki, Thessaloniki, Greece; 2 King’s College London, Institute of Psychiatry, Psychology and Neuroscience and Parkinson Foundation Centre of Excellence, King’s College Hospital, London, UK; 3 Department of Neurology, Technical University Dresden, Dresden, Germany; 4 Third Neurological Clinic, G. Papanikolaou Hospital, Thessaloniki, Greece; 5 Department of Neurology, Hippokration Hospital, Thessaloniki, Greece; 6 Department of Electrical Engineering and Computer Science/Department of Biomedical Engineering, Khalifa University of Science and Technology, Abu Dhabi, UAE

**Keywords:** Neurology, Signs and symptoms, Engineering

## Abstract

Fine-motor impairment (FMI) is progressively expressed in early Parkinson’s Disease (PD) patients and is now known to be evident in the immediate prodromal stage of the condition. The clinical techniques for detecting FMI may not be robust enough and here, we show that the subtle FMI of early PD patients can be effectively estimated from the analysis of natural smartphone touchscreen typing via deep learning networks, trained in stages of initialization and fine-tuning. In a validation dataset of 36,000 typing sessions from 39 subjects (17 healthy/22 PD patients with medically validated UPDRS Part III single-item scores), the proposed approach achieved values of area under the receiver operating characteristic curve (AUC) of 0.89 (95% confidence interval: 0.80–0.96) with sensitivity/specificity: 0.90/0.83. The derived estimations result in statistically significant ($p<0.05$) correlation of 0.66/0.73/0.58 with the clinical standard UPDRS Part III items 22/23/31, respectively. Further validation analysis on 9 de novo PD patients vs. 17 healthy controls classification resulted in AUC of 0.97 (0.93–1.00) with 0.93/0.90. For 253 remote study participants, with self-reported health status providing 252.000 typing sessions via a touchscreen typing data acquisition mobile app (iPrognosis), the proposed approach predicted 0.79 AUC (0.66–0.91) with 0.76/0.71. Remote and unobtrusive screening of subtle FMI via natural smartphone usage, may assist in consolidating early and accurate diagnosis of PD.

## Introduction

Parkinson’s Disease (PD), the second most common neurodegenerative disease after Alzheimer’s^1^, affects millions of older adults worldwide who face a wide variety of motor and non-motor symptoms during the progression of the disease^2^. The underlined pathology of PD involves the loss of dopaminergic neurons in a basal ganglia structure of the human brain (i.e., substantia nigra) and the presence of a widely distributed Lewy body containing, alpha-synuclein protein in the brain^3^, substantiating a prodromal period of PD with the presence of non-motor symptoms related with the olfactory system and the gut^4^. The standard PD screening is often made by a health care physician, usually a neurologist, who assesses the patient clinically and sometimes supported by patient related outcome measures within a limited time frame of about 15–30 min. Such consultations often spaced out 6 months intervals is beset with a range of problems leading to non-declaration of symptoms and possibility of sub-optimal care. The latter are caused by the short clinical consultations (often < 15 min)^5^, lack of use of objective clinical scales to assess the patient, such as the Unified PD Rating Scale (UPDRS)^6^, subjective assessments and ‘missed out’ symptoms and providing a ‘snapshot’ of the patient’s condition, rather than a continuous assessment^7^. Consequently, medication changes may be erroneous or unnecessary, whereas, accurate outcome measures are also crucial for research and clinical trial of drugs.

The motor syndrome of PD appears to be largely driven by reduced availability of dopamine at a striatal level linked to the degeneration of dopaminergic neurons in the substantia nigra pars compacta^3^. Motor symptoms evolve with time, being subtle and mild in early motor stages and advancing to progressive bilateral involvement with impairment of gait, balance, as well as overall mobility complicated by tremor, dyskinesia and fluctuations^8^. Recent work suggests that the subtle motor dysfunction of PD is evident even in the early prodromal stage before a clinical diagnosis of PD is made^9^. Detection of such impairments can lead to earlier diagnosis and implementation of management strategies earlier rather than late and current views support no advantage in starting treatment late in PD^10^. Furthermore, early detection may also help patients be available and ready for trials of new neuroprotective agents or indeed be treated with such drugs or strategies should this become available in the near future. Actually, it has been widely speculated that the failure of a large number of neuroprotection linked molecules in clinical practice, in spite of benefits in-animal models, is due to delayed intervention and the failure to detect PD early.

As detailed above, the standard medical practice in PD diagnosis, requires years of expertise from the physicians, lacks of accessible objective measures (which can improve treatment outcomes^11^) and is of low initial diagnostic accuracy^7^, increasing the risk of misdiagnosed early PD cases. Hence, there is an increasing need for quantitative assessment of PD using widely accessible Information and Communication Technology (ICT)-based tools that can assist the screening of PD high-risk population and remote monitoring of the PD symptoms’ status. Additionally, the wide and dense, in terms of frequency, usage of mobile devices can capture large-scale multi-modal habitual data, reflecting the severity of PD symptoms arising from human-mobile interaction. Research study mPower^12^, was the first large scale remote smartphone-based PD-related research effort that recruited participants, who self-reported their health status and were suggested to perform scheduled digitized tests for data collection towards remote PD screening. In spite of the big number of recruited subjects (9000), a dropout rate of 90% revealed that active data collection is not suitable for high-fidelity long term monitoring and participants are possibly subjected to the Hawthorne effect^13^. The latter could be addressed via passive capturing of users’ data during their natural use of smart devices. Toward this end, the remote smartphone-based research study HopkinsPD^14^ adopted a hybrid active-passive way of data capturing; subjects were asked to provide active and passive data via smartphone and self-reporting the demographics, the health status, and the medication dose per day. Researchers acquired  200 hours of passive data recordings per subject, including sensors’ data from accelerometer and GPS to measure movements, whereas active tests resulted in 35 recordings per subject. Nevertheless, the dominantly selected features for detecting impairment caused by PD arose from the active data collection and not the passive capturing, due to the high noise level induced in the sensors during the daily activities.

The findings of the latter remote human studies revealed the need for an unobtrusive study design towards long term adherence and simultaneous capturing of appealing data modalities, processed with tailored methods, to extract insightful data patterns for potential clinical adoption. User interaction with smartphones includes the dense activity of typing on touchscreens, which involves successive upper extremity movements of hands and fingers with coordination and subject’s attentive usage to the task. PD cardinal motor deficit (i.e., bradykinesia, rigidity, tremor, and/or postural instability) affects the body movement coordination^15^ and researchers linked it to keystroke dynamics (the sequences of timing information associated with keystroke presses and releases). The latter is an appealing source of information, since fine-motor impairment (FMI) has also been evident in the prodromal phase of PD^16^. Gallego et al^17^ introduced and validated the concept of analyzing physical keyboard typing data for PD detection and discrimination between PD patients and healthy controls. Their method exploited the key press hold time (HT) and achieved area under the receiver operating characteristic curve (AUC) of 0.83, when users typed in-the-clinic, and 0.76 AUC, when typed in-the-wild (e.g., at home) setting. Moreover, a statistically significant correlation of 0.50 was found between HT and the total UPDRS Part III score, including, though, UPDRS Part III items that are irrelevant to the FMI.

In our recent effort^18^, we have linked keystroke typing on smartphone touchscreen with an enriched feature vector, to describe keystroke variables (i.e., flight time (FT), HT, key pressure), achieving 0.92 AUC on the PD detection in-the-clinic setting and 0.62 correlation of the keystroke features with the UPDRS Part III single-item scores of the upper-extremity. This approach, provided a granular quantification of the severity footprint of the FMI. Therefore, in a subsequent analysis^19^, the best performing features were fed into a random forest regression algorithm to estimate specific UPDRS Part III single-item scores, aiming at extracting a specific relationship between the keystroke features and the FMI symptoms, achieving up to 0.83 correlation with the UPDRS Part III Single Item 22 (rigidity-related) in-the-clinic setting. In the case of remotely and unobtrusively acquired typing data in-the-wild, the produced touchscreen-based symptoms estimates showed good discrimination performance (0.84 AUC) on a self-reported cohort.

**Figure 1:**
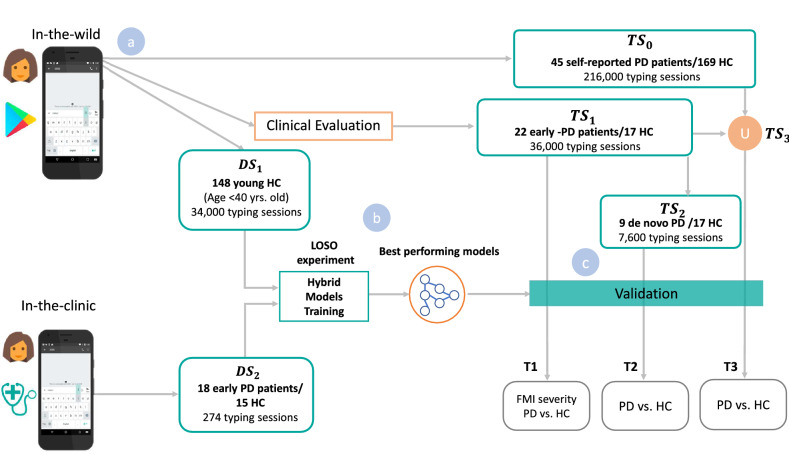
Overall study pipeline. (**a**) A training data-set arose in-the-clinic ($DS_2$) and used for training of the hybrid model along with a second training set ($DS_1$) arose from in-the-wild typing data remotely collected. (**b**) A hybrid model training using $DS_2$ and $DS_1$ produce best performing model for FMI estimation. (**c**) Separate data sets captured in-the-wild were used as test data for three test scenarios (T1, T2, T3); T1 correlates the estimated FMI severity with the clinical ground-truth, and the ability to classify early PD patients vs. HC in a test dataset ($TS_1$), T2 examines the classification performance to classify de novo PD patients vs. HC in a validation set ($TS_2$), a subset of $TS_1$, T3 examines the classification properties of the hybrid model in a large self-reported validation set, from the union of $TS_1$ and $TS_0$ ($TS_3$) against the subjects’ self-reported health status of being PD or not.

From the aforementioned, we explore, here, the hypothesis that touchscreen typing data acquired both in-the-clinic and in-the-wild settings, when combined with deep learning^20^, could provide a novel tool for the efficient remote screening of Parkisonian subtle FMI. Deep learning has been previously shown to be highly effective in extracting useful representations from high dimensional information, like images or temporal data. In PD screening, deep-learning algorithms can detect PD from MRI^21^, tremor from accelerometer^22^ and voice degradation from voice signals^23^. Extending this here, we show that deep learning can be leveraged to quantify touchscreen typing-based indices, from data captured remotely, passively and in-the-wild, that are strongly correlated with the FMI clinical scores and provide efficient screening of PD. A description of the development and evaluation process of the proposed method is depicted in Fig. 1 for the proposed hybrid model optimization and validation. In order to identify the severity of FMI, by merging the knowledge from the data captured both in-the-clinic^24^ ($DS_2$) and in-the-wild training dataset ($DS_1$), the hybrid model was optimized, targeting the highest correlation with the upper extremity UPDRS Part III single-item scores of 22/23/31 that express FMI severity. Other UPDRS III items could also measure Brady-/Hypokinesia (e.g., 24 and 25), but excluded hereby, since these items evaluate gross motor movements not efficiently reflected in the finger actions needed for typing. In order to test the scaling ability along with the symptoms severity and diagnostic properties of the hybrid models, three validation scenarios are employed. The first validation scenario (T1) evaluates the correlation of the estimated FMI severity with the clinical ground-truth, and the ability of the hybrid models to classify early PD patients vs. HC in a test dataset ($TS_1$) produced by 39 clinically examined subjects (22 early PD patients/17 healthy controls). The second validation scenario (T2) examines the discrimination performance of the method on the de novo PD vs. HC in a validation set ($TS_2$) (subset of $TS_1$), consisting of the 9 de novo PD patients and 17 HC, in order to examine whether the hybrid model can be used to detect PD on the drug naive phase. The third validation scenario (T3) examines the classification properties of the hybrid model in a large self-reported test set ($TS_3$) against the subjects’ self-reported health status of being PD or not.

## Results

### Hybrid model optimization

The estimated FMI severity from the trained hybrid model is expressed via the parameters dRSi, dAFSi, DBSi that correspond to Rigidity, Alternate Finger Tapping, Brady/Hypo-kinesia, respectively. The latter exhibited statistically significant (p < 0.001) correlation (std) of {0.56 (0.02), 0.76 (0.04), 0.64 (0.02)} with the FMI severity UPDRS Part III single items 22/23/31. This approach performed more efficiently when compared with the FMI severity estimations derived from networks solely trained in-the-clinic, i.e., {0.50 (0.11) 0.71 (0.08), 0.58 (0.09)}, accordingly.

### FMI severity estimation in T1 scenario

**Figure 2:**
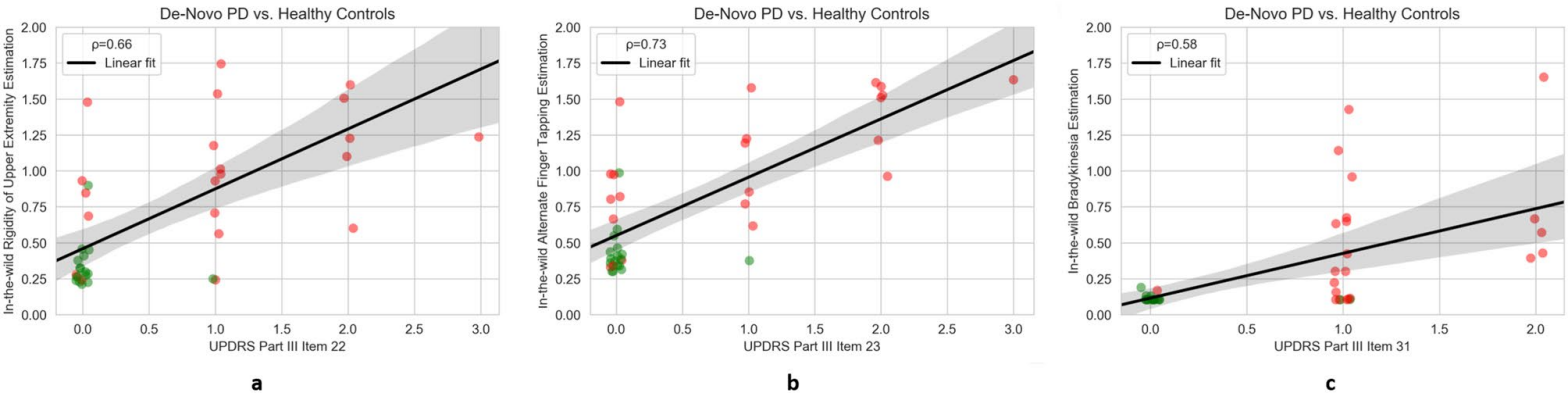
Estimated indices vs. motor clinical standards on the $TS_1$ dataset. The figure shows the relationship between UPDRS-III Part III single-item scores and the produced typing-based indices from typing sessions in-the-wild. Green dots represent the healthy controls and red dots represent the PD patients. Correlation coefficients between predictions and scores are significant (p < 0.001) in all three cases of 22 (**a**), 23 (**b**), 31 (**c**). The black line shows a linear fit and the grey area the confidence interval of the fitted line.

From the total typing sessions (36,000), 12,000 of them consisted of enough keystrokes ($\ge\, 40$) to be used in the validation analysis. The average age of the 22 early PD patients/17 Healthy Controls was 58.6/54.6 years, 73%/59% of the subjects were males, and were tested for being demographically matched. Additional subjects’ demographics are tabulated in Table 1. A correlation analysis between the subjects’ upper extremity UPDRS Part III single items 22/23/31 and the hybrid model predicted parameters, i.e., dRSi, dAFSi, DBSi, exhibited correlation of 0.66/0.73/0.58, respectively. These results show that the proposed hybrid approach outperforms the previous method developed^19^, which achieved corresponding correlations of 0.64/0.58/0.55, respectively. In Fig. 2, the predicted dRSi, dAFSi, DBSi values per subject and the respective clinical items are depicted with colored dots highlighting the different populations (red for PD patients, green for HC). As it is clear from Fig. 2 a strong relationship between the derived typing-based indices and the severity of the symptoms as the scores increase is noticeable.

**Table 1:** Baseline characteristics of $TS_1$ cohort. The two groups are reasonably matched in terms of demographics as no significant differences (p < 0.05) are observed (two-sided Mann–Whitney *U* test). With the exception of clinical characteristics (UPDRS Part III score), ^a^Avg. Levodopa Equivalent Daily Dose (LEDD) concerns only PD patients under treatment (n = 13). Additional information, especially for de-novo PD patients can be found in Supplementary Table S1. *N.A.* not applicable, *sig.* significant, *n.s.* non-significant. SU1: SU < 6 months; SU2: SU $\in$ [6–12] months; SU3: SU > 12 months.

Characteristics	Early PD patients	Controls	Statistical significance
n (total n = 39)	22	17	N.A.
**Demographics**			
Women # (%)	6 (22%)	7 (41%)	n.s. (p = 0.57)
Men # (%)	16 (78%)	10 (59%)	n.s. (p = 0.57)
Avg. age, years (std)	58.6 (8.4)	54.6 (9.4)	n.s. (p = 0.07)
Subjects #/# who completed Education Level H/U	9/13	4/13	n.s. (p = 0.83)
Smartphone usage (SU) (SU1/SU2/SU3)	2/1/19	0/0/17	n.s. (p = 0.29)
**Clinical characteristics**			
Avg. disease onset, years (std)	2.5 (1.6)	N.A.	N.A.
Avg. UPDRS Part III score (std)	19.7 (14.6)	1.3 (2.1)	Sig. (p <0.001)
PD patients #/# under treatment/de novo	13/9	N.A	N.A.
Avg. LEDD^a^, mg (std)	395 (154)	N.A.	N.A.
Avg. valid recordings (std)	195 (511)	269 (511)	N.A.

### Remote screening of PD in T1 scenario

**Figure 3:**
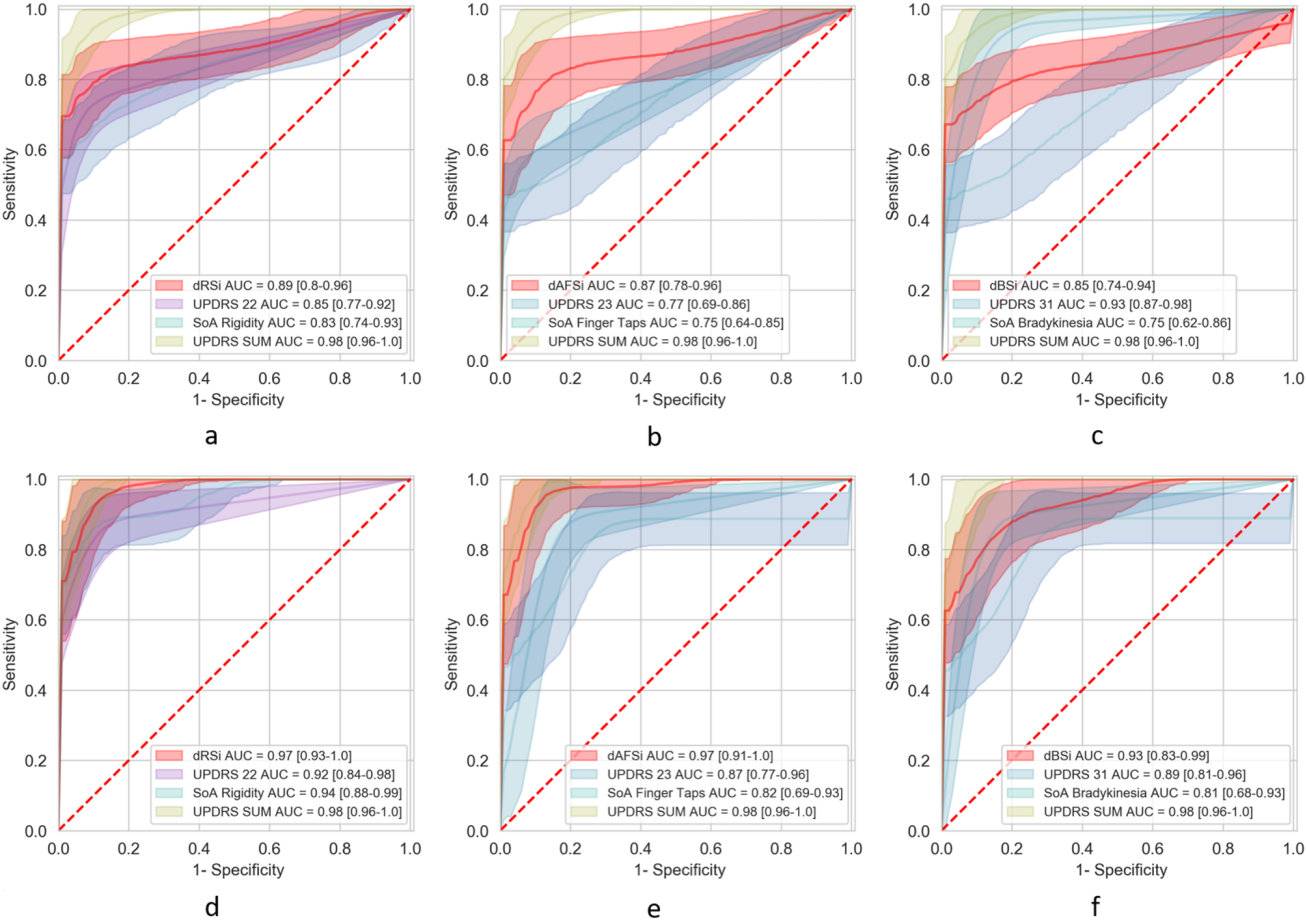
Prediction of Parkinson’s disease classification. Comparison of Receiver Operating Characteristics (ROC) curves in (**a**)/(**b**)/(**c**) for the produced dAFSi/dRSi/dBSi vs. the state of the art (SoA) algorithms, UPDRS Part III single item 22/23/31 and sum of UPDRS Part III in the $TS_1$ cohort for early PD patients vs. HC classification, and the respective comparison curves in d/e/f regarding the de novo PD patients vs. HC classification. AUC values for models are shown with 95% CIs.

Receiver operating characteristics (ROC)-based performance of the indices derived from the $TS_1$ set under the T1 scenario (Fig. 1), are depicted in Fig. 3. In particular, the performance of the predicted dRSi, dAFSi, DBSi values per subject, when compared to the previous method^19^, the UPDRS Single Item score 22/23/31 and the sum of UPDRS Part III items, is depicted. Specifically, in T1 scenario, the dRSi was the best performing typing-based index, achieving 0.89 [with 0.83–0.97 95% confidence interval (CI)] area under the ROC curve (AUC), and with 0.90/0.83 sensitivity/specificity, whereas the UPDRS Part III items 22/23/31 achieved AUC of 0.84 (with 0.77–0.92 95% CI)/0.77 (with 0.69–0.86 95% CI)/0.93 (with 0.87–0.98 95% CI) with {0.94/0.73 ,0.94/0.60, 0.88/0.95} sensitivity/specificity per item, respectively. Moreover, the sum of UPDRS Part III items and the exhibited AUC values of 0.98 (with 0.96–1.00 95% CI) with 0.94/1.00 sensitivity/specificity, whereas the previous methods achieve lower classification properties achieving up to 0.83 AUC (with 0.74–0.93 95% CI) with 0.88/0.73 sensitivity/specificity, respectively. In order to examine the ability to perform remote passive screening we tested our indicators regarding the sensitivity at increasing level of specificity (see Table 2) whereas dAFSi, dBSi and dRSi performs 51%, 36% and 43% sensitivity, respectively, when specificity is 90% in the $TS_1$.

### Remote screening of PD in T2 scenario

ROC-based performance of the indices derived from the $TS_2$ set, consisting of 9 de novo PD patients (under no PD medication) and 17 HC (see Suppl. Materials for demographic characteristics) under the T2 scenario (Fig. 1), are depicted in Fig. 3. Specifically, the dRSi/dAFSi/dBSi exhibits AUC 0.97 (with 0.93–1.00 95% CI)/0.97 (with 0.91–1.00 95% CI)/0.93 (with 0.87–0.98 95% CI) with {0.87/0.90, 0.93/0.90, 0.82/0.90} sensitivity/specificity, respectively and achieve better diagnostic performance over the UPDRS Single item scores, with the best (item 22) achieving ROC AUC 0.92 (with 0.84–0.98 95% CI) with 0.88/0.87 sensitivity/specificity. The dAFSi is of similar performance with the diagnostic properties of the sum of the UPDRS Part III score, which achieves AUC score of 0.98 (with 0.96–1.00 95% CI) with 0.94/1.0 sensitivity/specificity.

**Table 2:** Sensitivity is presented with 95% CIs. Bold text indicates the highest sensitivity among the models at each specificity and test set.

	Specificity		
	70%	80%	90%
***TS***_**1**_ **(Clinically Examined PD patients vs. HC)**			
dAFSi	**95% (94.0–98.9)**	**90% (88.0–93.9)**	**51% (23.0–70.9)**
dBSi	90% (82.0–100.0)	69% (40.0-98.9)	38% (12.0–84.9)
dRSi	92% (83.9–98.9)	85% (73.0–94.0)	43% (29.9–68.9)
***TS***_**2**_ **(Clinically examined de novo PD vs. HC)**			
dAFSi	**96% (93.9–98.9)**	**96% (93.9–98.9)**	**93% (89.0–93.9)**
dBSi	92% (83.0–100.0)	86% (72.0–100.0)	82% (71.0–94.1)
dRSi	**96% (93.8–99.0)**	92% (84.0–98.9)	87% (77.0–93.9)
***TS***_**3**_ **(Self-reported PD patients vs. HC)**			
dAFSi	**77% (75.0-78.0)**	**67% (60.0-72.0)**	**40% (20.9-51.0)**
dBSi	69% (63.0-79.0)	54% (31.0-77.1)	30% (0.05-70.9)
dRSi	77% (70.0-84.0)	53% (38.0-66.0)	38% (28.0-50.0)

### Remote screening of PD in T3 scenario

Regarding ROC-based performance in the $TS_3$ where subjects are classified against their self-reported label, dRSi, dAFSi, dBSi achieve AUC 0.79 (0.66–0.91 is the 95% CI)/0.78 (0.62–0.90 is the 95% CI)/0.75 (0.69–0.76 is the 95% CI) with {0.76/0.71, 0.75/0.68, 0.75/0.73 sensitivity/specificity}. The previous methods^19^ achieved lower diagnostic performance from the proposed approach and AUC 0.74 (0.59–0.89 is the 95% CI), 0.71 (0.55–0.85 is the 95% CI), for estimation of UPDRS 22/31 respectively. When targeting the specificity level of 90% (see Table 2) the dAFSi, dBSi,dRSi achieves 40%, 30%, 38% sensitivity, sustaining, even in a self-reported cohort, good sensitivity levels in a highly unobtrusive concept. The resulted lower diagnostic properties that these indices exhibit on the $TS_3$ cohort can be explained from the different factors that may cause fine-motor impairment, such as sleep inertia^25,26^, depression^27^ and arthritis^28^.

**Figure 4:**
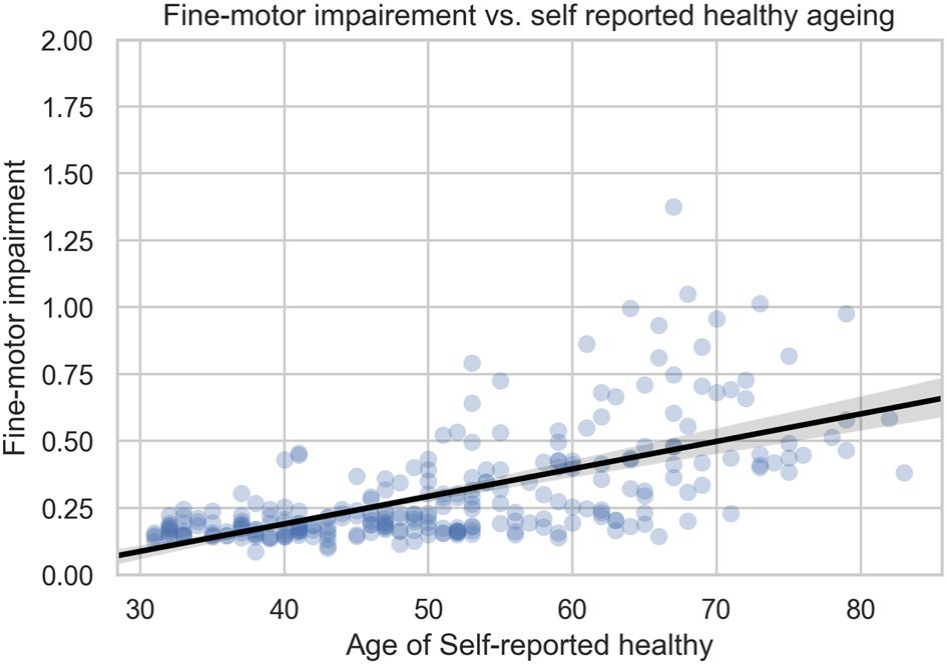
Estimation of fine-motor impairment for all healthy subjects across all self-reported age. Statistically significant (p < 0.001) Pearson’s correlation coefficient of 0.62 was computed between the mean estimated FMI per subject and the age variable.

Moreover, Logistic Regression tests showed that the produced indices are the only statistically significant variables, when used as independent variables with education, age, gender, years of usage, and the health status used as dependent ones in the T1, T2, T3 scenarios (tests results are included in Supplementary Materials). Moreover, since the results of the FMI severity estimation proved to be robust in-the-wild, we further investigated the relation of the estimated FMI with the age of the healthy population (self-reported HC subjects) in-the-wild cohort, as depicted in Fig. 4. A correlation of 0.62 between the age and the estimated FMI was found in self-reported HC, which is in agreement with previous research suggesting that aging deteriorates the spatial coordination of fine-motor movements, and especially reduces movement speed, increases variability and imposes coordination difficulties^29,30^.

## Discussion

This study proposes a remote passive screening tool that showcases the ability to quantify subtle FMI based on the analysis of typing on mobile touchscreens using deep learning. The results of the study were derived by comparing the diagnostic properties validated on different datasets captured in-the-wild, forming an ecologically valid way of objective screening of FMI. Specifically, in the case of clinically confirmed and examined subjects, the best performing typing index achieves diagnostic performance comparable with UPDRS Single Item scores of the same population, in efficiently classifying early PD patients from HC. The proposed remote method overcomes the quantization error that the UPDRS scale induces^7^, by outputting a continuous and reliable score for the FMI severity detection. Moreover, the subjective nature of FMI detection within the clinical PD examination^31^, is addressed by objective remote measures proposed hereby. The latter provide increased FMI time-resolution monitoring between the transition period from HC to PD status, surfacing subtle changes that could lead to a timely-efficient clinical consultation.

Regarding the correlation of the proposed approach with the clinical ground-truth, the maximum correlation coefficient value (0.73) among all metrics under scrutiny was found between the severity prediction of AFT, via the dAFSi, and the respective clinical item (UPDRS Part III item 23), as shown in Fig. 2, projecting the effect of brady/hypo-kinesia to the FMI. This is further justified due to the AFT resemblance with the standardized clinical examination process^32^, that is PD patients tap their thumb with the index finger in rapid succession. It is noteworthy, that although body brady/hypo-kinesia (as described from UPDRS Part III item 31) is implicitly associated with FMI, the dBSi still yields a statistically significant correlation value (0.58), showing the efficiency of the proposed approach to capture all related FMI information, in order to maximize its detection accuracy.

In all three presented test scenarios (T1, T2, T3), the estimated sensitivity/specificity values (Table 2) indicate that the proposed method can be exploited for reliable passive remote screening of PD, with low false-alarm performance. Moreover, in the T2 scenario incorporating de novo PD vs. HC classification, dRSi and dAFSi achieve similar diagnostic properties to the total clinical UPDRS Part III examination, possibly due to the fact that FMI may be present in the early onset phase^16^. This shows that the proposed approach is suitable to assist early PD detection.

FMI estimation shows strong correlation with the clinical ground truth (clinical assessment of rigidity of upper-extremity and brady/hypo-kinesia) of symptoms that cause muscle stiffness and slowness of movement, as well as decreased movement amplitudes. Encouragingly, this is also reflected in the Convolutional Neural Networks (CNN) activations^33^ for FT and HT typing inputs, depicted in Fig. 6 (HC/PD: top/bottom panel). Specifically, in Fig. 6a, the activation of CNN for the HC case peaks between 20th-30th samples (blue-shaded area); this complies with the lower HT values (Fig. 6b) and reduced variance of rhythmic typing, as seen by the concentrated FT values (Fig. 6c), for the same sample range. This is expected as this is an HC case. In the case of PD, though, the activation of CNN peaks in two sample areas, i.e., 1st-8th samples (orange-shaded area) and 15th-30th samples (blue-shaded area)(Fig. 6d), where higher and more dispersed HT (Fig. 6e) and FT values (Fig. 6f) are observed. These results showcase the adaptivity of the CNN functionality to capture the most informative input parts, justifying the effectiveness of the proposed approach when compared to the window-based FMI estimation methods^19^ (Fig.3).

Being consistent with the findings of our method regarding HC vs. PD classification, yet with less AUC of 0.76 (sensitivity/specificity of 0.73/0.69) and moderate correlation of 0.34 with the total UPDRS Part III score, Arroyo et al.^17^ examined the diagnostic properties of an HT-based index produced by typing on a physical keyboard in-the-wild, as an alternative FMI estimation method. In the same line, Iakovakis et al.^19^ used HT- and FT-based features from keystroke dynamic data on a touchscreen keyboard to identify the bradykinesia and rigidity symptoms; however, with lower diagnostic performance, as it can be seen in Fig. 3, in all validation scenarios. All these efforts denote that the value of the underlined diagnostic information that lies in the keystroke dynamics regarding PD detection. The increased detection performance of the current approach (Figs. 2, 3) justifies further the value of this research path, even when the touchscreen typing data arise in-the-wild.

**Figure 5:**
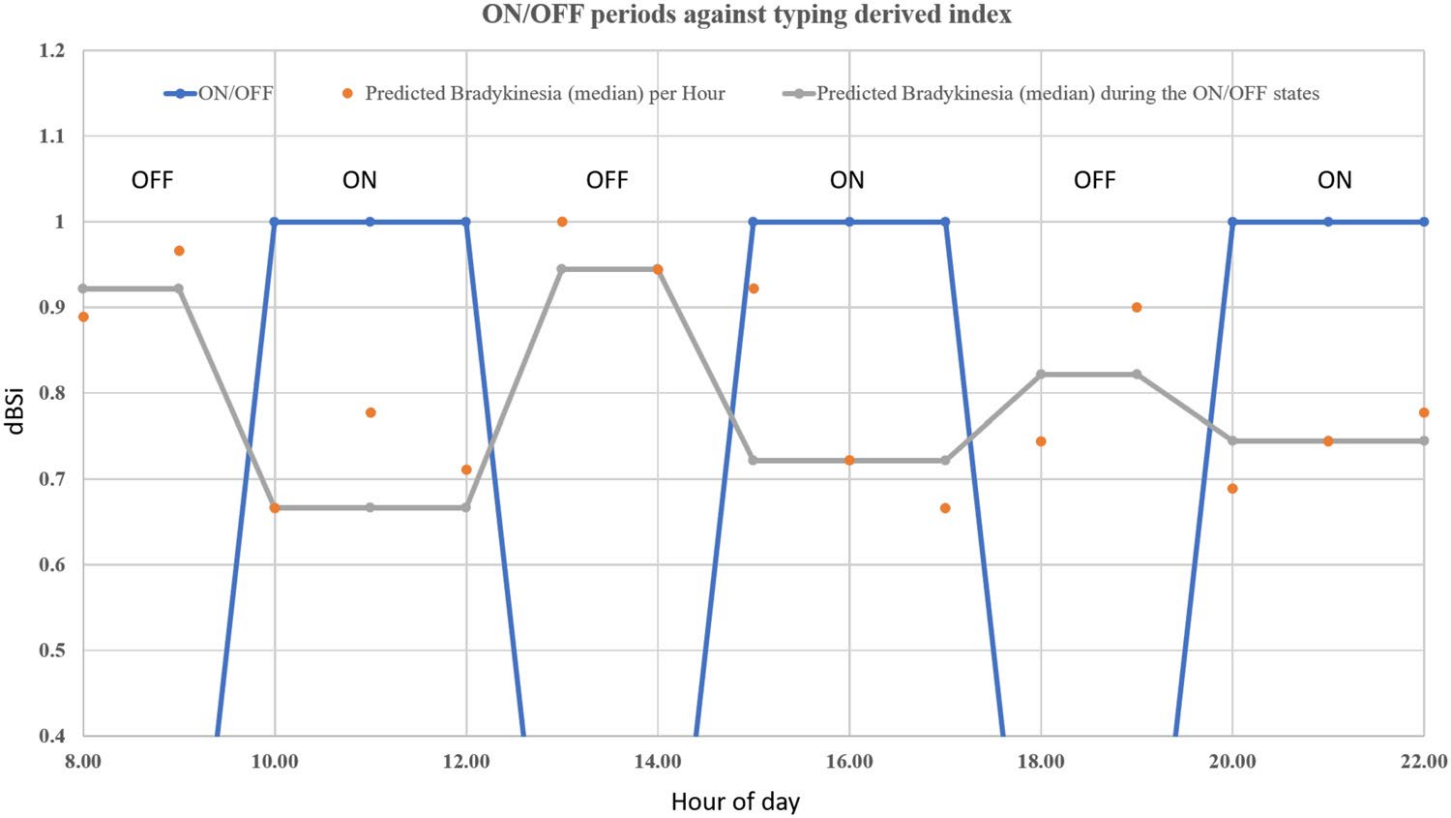
Median dBSi, in absolute values, is calculated across hours, as derived from a $TS_1$ participant, with the respective median index during these periods (gray lines). The nominal ON/OFF periods, as derived from the physician (see Methods), are projected in the 1/0 (blue dotted line) interval for comparison.

Interestingly, one potentiality that was identified in the proposed approach was a very preliminary finding for a case of a specific PD patient in $TS_1$. More specifically, this PD patient belongs to the patient’s group under medication (Table 1), and was used as a case study in shedding light on the potential application of the developed method on daily monitoring of PD medication. Currently, the latter relies on patients’ self-reporting of the ON/OFF motor states, usually in an outpatient clinic scenario drawing on recollections. Diary-based observations are also occasionally used and such approaches are fraught with inaccuracy if data and facts generated. However, ICT-based research results in detecting these periods^34^ are usually based on additional hardware or at-home device monitoring to monitor subjects’ drug response over time. Hereby, the detection ability of the hybrid models to capture the ON/OFF alternations in an advanced PD patient from our cohort was explored. The ground-truth ON/OFF periods across the time-range of 08:00–22:00 were identified. For each ON/OFF period the values of 1/0 were assigned, respectively, with transition period of 30 minutes, shown as blue trapezoids in Fig. 5. In the latter, the nominal FMI predictions (orange dots) and their median value (gray line) across the same time-range are superimposed. When comparing the ground-truth ON/OFF periods with the predicted median values a statistically significant correlation ($p<0.05$) of − 0.90 was found. Despite the limited conclusions that could be drawn due to the examined single-case, further insights in cases of monitoring of PD, clinical trials and precision medicine, as an objective comparison of the drug efficacy, could be obtained, regarding the drug-response of FMI^35^. The latter is consistent with the findings of other study^36^ who also find that typing data from physical keyboard (as an alternative FMI estimation mechanism) allows for detection and prediction of drug response. Further investigation could lead to a system capable to adjust drug infusion rates in apomorphine^37^ and duodopa pumps^38^ based on the patients’ fine-motor state. However, the preliminary findings require a larger cohort with advanced PD cases, not present in the current study, with consistent reporting of the medication, for further justifications of these preliminary, yet interesting, finding.

Despite the promising results, our study has several limitations. Firstly, the overall size of the clinically evaluated subjects in-the-clinic is small for deep learning, although the use of the hybrid model training with the CNN autoencoder was suitable to amalgamate this issue. Similarly, the clinically assessed $TS_1$, and its subset $TS_2$, comprises of a small number of participants. This is due to the variety and cost of processes involved in subjects’ on-site recruitment and physicians’ engagement for a concrete clinical examination. Nevertheless, the dense data contribution, the narrow CI presented for AUC scores for the deep learning-based typing indices (Fig. 3), and the resulted metrics correlation with the UPDRS Part III Single item scores, imply a promising use of the method. Obviously, a significantly larger $TS_1$ and $TS_2$ may enable more accurate deep-learning models to be trained and evaluated, contributing to more refined subtle-FMI detection models. Another possible limitation of this study is the validity of the users’ self-reported demographics, and the absence of clinical characteristics in the $TS_3$ setting. However, the existence of the clinically examined cohort and the discrimination performance of the proposed indices justify the screening ability of the method. Moreover, from an overall perspective towards prodromal PD detection, a wide non-motor spectrum of symptoms in the prodromal phase should be taken into consideration, in parallel to the subtle motor symptom examined here. The ecologically valid detection of one or more related non-motor symptoms, i.e., depression, sleep, anxiety, postural dizziness and pain, could be fused in a multi-modal research effort towards early PD detection. The latter study-design is currently explored in the context of the ongoing research project i-PROGNOSIS (http://www.i-prognosis.eu/, that formalizes a behaviour-related vector including motor and non-motor symptoms to enhance the promising diagnostic properties of the current typing-based metrics.

Overall, we proposed a user-centric, ecologically valid way of behavioral monitoring tailored to passive screening, allowing for long-term adherence and high data fidelity, increasing the added value of the smart devices. Using data drawn from in-the-clinic and in-the-wild acquisition settings, our hybrid model provides satisfactory performance in detecting subtle FMI. Compared to current approaches, our method performs similarly to the clinical evaluation allowing for densed remote sensing of FMI time-evolution. The neuropathological process that leads to PD raises the intriguing possibility that environmental substances can trigger pathogenesis, and, hence, the FMI estimation can be used for longitudinal screening of people at high risk of developing PD^39^. Probing further, our work suggests avenues of future research in other diseases causing FMI, like arthritis^28^, diabetes, where the severity of FMI increases at altered glucose levels^40^, or even cognitive impairment^41^, when compared to healthy ageing population. Specifically, our method applies in cases of early screening and monitoring of Alzheimer’s disease (AD) or Mild Cognitive Impairment^41^, since sensory and motor changes may precede clinical manifestations of AD by 10 or 15 years^42^ prior to the diagnosis. This is important as, the validated clinical AD diagnosis tests are not sensitive enough in detecting deviations from healthy-ageing trajectory, and suffer from intrinsic biases^43^. Moreover, progressive slowing of fine motor dexterity has been associated with cognitive deficits^44^, which also allow the investigation of the current method for depressive tendency screening^27, 45^ and mood alterations associated with mental health^46^. In conclusion, remote passive screening of subtle fine-motor symptoms via deep learning-based smartphone typing information reveals great potential in estimating subtle FMI and its severity via natural use of smartphone. Clearly, the objectiveness and efficiency of the proposed methods contributes to early PD detection efforts.

## Methods

### Data capturing

The custom-made keyboard of the mobile iPrognosisapp https://play.google.com/store/apps/details?id=com.iprognosis.gdatasuite, developed for the Android Operating System (OS) by three authors (D. I., S. H. and V. H.), was used for capturing all keystroke-related data of the study. The capturing process followed a privacy-aware manner, capturing the keystroke dynamics only and not the typed content *per se*. For each typing session (with at least one key-tap), the captured keystroke data were stored in a JSON format file and indexed as an entry in a local SQLite database. The acquired local database entries were daily uploaded to a remote Microsoft Azure cloud-based database and each entry was accompanied by a unique coded user ID, to sustain data privacy and ensure GDPR^47^ compliance. Regarding the datasets that included clinical evaluation (Fig.1), each coded user ID was used by the clinicians, via the OpenClinica platform (https://www.openclinica.com/), to associate the subject’s data with his/her clinical examination outcomes and demographic information.

With regards to the data captured in-the-wild, they were drawn from subjects coming from seven countries across EU and Australia, passively providing de-identified keystroke dynamics data in a remote manner via the iPrognosis app. All contributing subjects, provided electronic informed consent during the iPrognosis installation on their smartphone, by digitally signing a dated consent form, preserving the right to withdraw from the procedure at any time, and even request the deletion of their collected data so far.

### Data splitting

For the development and testing of the proposed approach, the acquired data were split in different development and testing datsets, as described in details below. Note that 40 keystrokes was set as the minimum threshold to consider a typing session valid in all datasets.

#### Development datasets ($DS_1,\; DS_2$)

The development dataset $DS_1$ (Fig. 1) consists of 34,000 typing sessions with keystroke dynamics drawn from subjects with age $\le\, 40$ years (self-reported).

The $DS_1$ dataset belongs to the in-the-wild category and the related keystroke dynamics were only used for unsupervised pre-training of the neural network parameters.

The development dataset $DS_2$ (Fig. 1) consists of keystroke dynamics from 33 demographically matched subjects (18 early PD patients and 15 HC) and belongs into the category of data in-the-clinic. These subjects underwent clinical examination by neurologists, and existence of any FMI was grounded by their UPDRS Part III single-item scores 22/23/31 that express rigidity of upper extremity/alternate finger tapping/general body bradykinesia-hypokinesia, respectively. Note that in the clinical evaluation, both hands are examined and separate UPDRS Part III scores are derived for each hand. However, the right-hand scores were only adopted here, as all subjects of the $DS_2$ were right-handed and used their main hand during typing. Subjects typed on a smartphone touchscreen 274 typing sessions in total (up to ten text-excerpts each), following a controlled protocol. A more detailed description of the $DS_2$ can be found in our previous work^18,24^. The captured keystroke dynamics were used as input variables and the respective FMI scores were used as labels for the supervised fine-tuning of the network.

#### Testing datasets ($TS_0,\; TS_1,\; TS_2,\; TS_3$)

Four data sets coming from in-the-wild cohort, i.e., $TS_0,\; TS_1, \;TS_2,\; TS_3$ (Fig. 1), were formed for the testing of the proposed approach. Specifically, $TS_0$ includes keystroke dynamics data from 214 subjects, who self-reported their demographics information via the iPrognosis app, providing 216,000 typing sessions.

$TS_1$ dataset was formed in the same manner as the $TS_0$, yet the related subjects underwent clinical examination, as in $DS_2$ (Fig. 1); however, their data still were gathered under the in-the-wild scenario. $TS_1$ consists of the keystroke dynamics (36,000 typing sessions) and the detailed clinical examination data (as reported by neurologists) from 39 subjects (PD/HC: 22/17; demographics are tabulated in Table 1).

$TS_2$ dataset is a subset of $TS_1$ (Fig. 1), containing keystroke dynamics data (7,600 typing session) drawn only from de novo PD patients and combined with the ones coming from the same HC as in $TS_1$ (de novo PD/HC: 9/17; demographics are tabulated in supplementary Table S1).

The union of $TS_0$ and $TS_1$ forms the $TS_3$ dataset (Fig. 1) that consists of keystroke dynamics data (252,000 typing session) from 253 subjects (PD/HC: 67/186, demographics are tabulated in supplementary Table S2).

#### Data input sequences

The sequences of hold time (HT) and flight time (FT) were used as data inputs to the proposed model. They are defined as $HT_n = t^{r}_{n}-t^{p}_{n}$, $n=1,2,\ldots,N,$ and $FT_n = t^{p}_{n+1}-t^{r}_{n}$, $n=1,2,\ldots,N-1$, where $t_n^p$ and $t_n^r$ are the timestamps of touchscreen press (*p*) and release (*r*) events of finger interaction with the mobile touchscreen, respectively. Before any data analysis, a conditional filtering process was applied to HT sequences, discarding deliberate long-presses that correspond to special actions, such as selection of accentuated letter and/or special characters; hence, an upper bound of 3s was applied to FT sequences, so to eliminate misleading data. Zero padding was applied to both HT and FT sequences to create two fix-length vectors of 100 elements per typing session, in case of session length $\le\, 100$.

#### Hybrid models development

The proposed hybrid models (one for each symptom of FMI) were developed in three sequential steps, as described below.

*Step 1: Representation learning.* One dimensional CNN-based autoencoders^48^ with two input channels (for HT and FT) were used to learn an efficient neural networks’ encoding mechanism for representing the keystroke dynamics. They consisted of sequentially connected convolutional layers with or without max-pooling layers. The CNN learnt the temporal characteristics of keystroke typing in-the-wild, using the $DS_1$ dataset via unsupervised training, where the goal was to learn the identity function, so the output being as similar as possible to the input. Through this process, the network learns the inherent structure of the keystroke dynamics and represents them via a limited number of features. The latter are used within a transfer learning process in the next step of the models development.

*Step 2: Fine-tuning.* An additional two-layer fully-connected network was sequentially added to the pre-trained networks and the final network was fine-tuned to estimate each symptom’s severity, i.e., rigidity of upper extremity, alternate finger tapping and general body bradykinesia-hypokinesia. The fine-tuning was realized via the training of regression models with leave-one-subject-out cross-validation, targeting network parameters optimization, by using as independent variables *FT* and *HT*, and as dependent ones the UPDRS Part III single-item scores.

*Step 3: Optimization.* The hybrid models were optimized in a subject level using $DS_2$, to approximate the scale of each symptom’s severity on each subject. The latter is the outcome of the average symptom’s severity estimation across the subject’s valid sessions, as the related data distribution per session was similar. This approximation was evaluated by using the Pearson’s correlation coefficient between each model’s prediction and the corresponding UPDRS Part-III Single Item score. Mean and standard deviation of the correlation coefficients were also computed for each set of the predictions for the different indices. Furthermore, the models were evaluated without the representation learning (Step 1), for evaluating the contribution of the latter to the overall performance.

The hybrid models’ output that best correlated with the clinical items, denoted as d-B/R/AF-Si for deep Bradykinesia/Rigidity/Alternate Finger Tapping Severity indices, respectively, were used for testing in-the-wild (see Testing scenarios below). Since the keystroke dynamics data coming from the in-the-wild cohort had increased variation in the session distribution per subject, the three quartiles (i.e., the 25th/50th/75th) of the subjects’ contribution were used, in order to reach a final FMI estimation per subject.

#### Implementation issues

In Step 1, kernel sizes of 3 and 5 were used for the convolutional layers with the sizes of filters ranging from 8 to 32 during the experiments. A 80–20% train-test split was made for the data during the training process, while the network was trained with back-propagation for 50 epochs with mini-batches of size 64, using the RMSprop^49^ optimizer, with a learning rate of $10^{-3}$ and optimized the mean squared error loss function. In the fine-tuning step (Step 2), 50 epochs with mini-batches of 32 were selected, whereas the same RMSProp optimizer^49^ as in Step 1 was adopted. The loss function that the network tried to optimize was the Mean Squared Error between the estimation and the ground-truth UPDRS Part III single-item scores. In the fine-tuning process, the network hyper-parameters were explored, i.e., the different number (i.e., 10, 20, 50, 100) of nodes of the fully connected layers and the number of layers of the pre-trained convolutional layers. The training experiments were implemented in Python 3^50^ and the model was trained and tested on a Microsoft Azure virtual machine (56 GB RAM with a NVIDIA Tesla K80 GPU).

### Testing scenarios (*T*1, *T*2, *T*3)

The first testing scenario (T1) evaluates the correlation of the estimated FMI severity with the clinical ground-truth, and the ability of the proposed hybrid models to classify early PD patients vs. HC in the $TS_1$ dataset. The second testing scenario (T2) examines the classification performance of the hybrid models on the de novo PD vs. HC classification, in order to examine whether the hybrid models can be used to detect PD the drug naive phase, using the $TS_2$ dataset. Finally, the third validation scenario (T3) examines the classification performance of the hybrid models in $TS_3$ dataset, against the subjects’ self-reported health status of being PD or not.

### Model explanation

**Figure 6:**
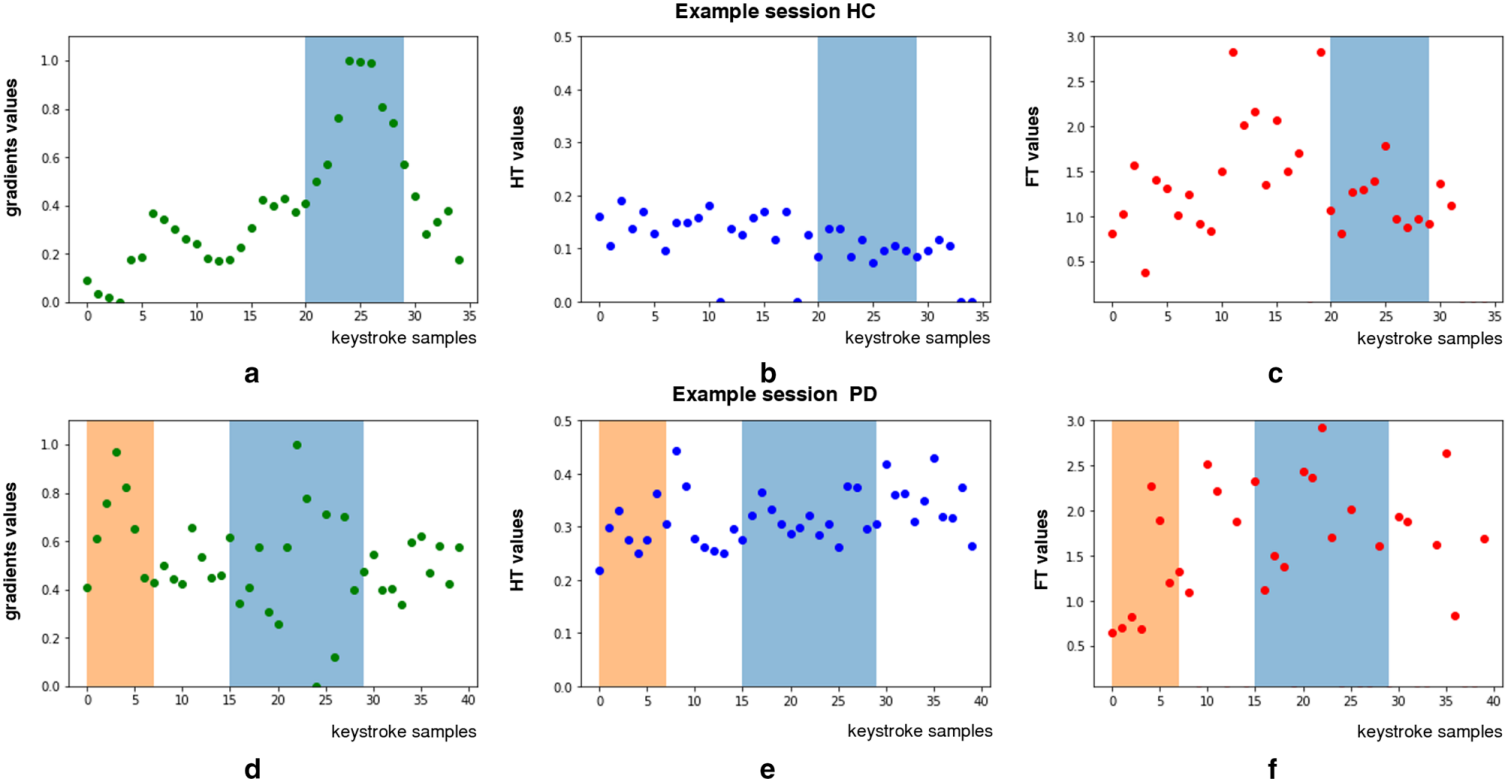
Attention maps of CNNs shown with respect to the input sequences of HT and FT (top/bottom panels for HC/PD of two cases from the TS1 dataset, respectively). Top panel-HC case: (**a**) activation of CNN, (**b**) HT values, (**cc**) FT values; Bottom panel-PD case: (**d**) activation of CNN, (**e**) HT values, (**f**) FT values. Each sample represents a datum of the keystroke typing session, whereas the shadowed areas (blue and orange) denote the sample range that the neural-network model is using to make the prediction for the typing session.

The attention map was used to understand which regions in the typing data mostly influenced the prediction process of the hybrid models, when predicting high or low FMI. We present the saliency maps obtained using a method^33^ that computes the gradient of output predictions with respect to the input, and altering it, in order to fit the typing data. These can be presented as increased values of one-dimensional heatmaps to output a visual indicator of the importance of any given region on the series of FT and HT (Fig. 6, providing a relevant explanation of the typing behaviour.

### ON/OFF periods detection

The association of the intra-day fluctuations of motor impairment, as caused by the medication, with the respective estimation of FMI was examined as a case-study for the PD subject of $TS_1$ dataset. This subject was selected, as he was the only one in the $TS_1$ with advanced PD; hence, the ON/OFF periods due to medication intake are evident in his everyday life activities. The ON/OFF periods were extracted from the hours that the clinician and patient reported as the most usual time for the L-dopa intake, with a linear transition of half an hour before and after the ON phase. Furthermore, the subjects’ estimated FMI data were aggregated for each hour of the day, and their median response over the ON/OFF hours was calculated for comparison (see Fig. 5).

#### Performance evaluation and statistical analysis

The performance of the proposed hybrid models was evaluated across five different random states of the networks. The FMI estimations were evaluated in the binary classification performance (PD vs. control), by estimating the area under curve (AUC) of the Receiver Operating Characteristics (ROC) curve. The ROC based performance was computed with Confidence Interval (CI) with 1,000 bootstraps. The sensitivity/specificity metrics were produced via searching a threshold on the prediction that satisfies the specificity levels of 70/80/90% (Table 2). The two groups of PD and controls (for the T1 ,T2, T3 testing scenarios), were tested in terms of demographics using a two-sided Mann–Whitney *U* test for the age, and Chi-squared test for the smartphone usage, gender, and education level. Moreover, logistic regression tests were used to test if the prediction of the subject status (PD or control) was influenced by the sex, age, years of education and smartphone usage. The corresponding results are reported in detail in supplementary Figs. S1, S2, and S3. Statistical analysis and performance evaluation have been made with scientific computing Python packages^50^.

### Ethics approval

All research protocols were approved by Ethik-Kommission an der Technischen Universität Dresden, Dresden, Germany (EK 44022017), Greece, Bioethics Committee of the Aristotle University of Thessaloniki Medical School, Thessaloniki, Greece (359/3.4.17), Portugal Conselho de Ética, Faculdade de Motricidade Humana, Lisbon, Portugal (CEFMH 17/2017), United Kingdom London, Dulwich Research Ethics Committee (17/LO/0909),Comité de Ética de la Investigación Biomédica de Andalucia, Spain (60854c5dbc58dda37b4730edb590a503edbd3572), Sunshine Coast Hospital and Health Service, Australia (41562 HREC/18/QPCH/266) and Studienzentrum der Prosenex AmbulatoriumbetriebsgesmbH an der Privatklinik Confraternitaet, Wien, Austria (002/2018). Recruitment and study procedures were carried out according to institutional and international guidelines on research involving adult human beings. Subjects held the right to withdraw from the study at any time, without providing any justification. All participants, including subjects with early PD, were capable to provide informed consent prior to their participation in the study.

## Supplementary information

Supplementary Information.

## Data Availability

All data generated and analysed during the current study are available from the corresponding author on a reasonable request.

## References

[1] Chaudhuri KR, Titova N (2019). Societal burden and persisting unmet needs of Parkinson’s disease. Eur. Neurol. Rev..

[2] Titova N, Chaudhuri KR (2018). Non-motor Parkinson disease: new concepts and personalised management. Med. J. Aust..

[3] Kalia LV, Lang AE (2016). Parkinson disease in 2015: evolving basic, pathological and clinical concepts in PD. Nat. Rev. Neurol..

[4] Klingelhoefer L, Reichmann H (2015). Pathogenesis of parkinson disease—the gut–brain axis and environmental factors. Nat. Rev. Neurol..

[5] Playfer JR, Hindle JV (2008). Parkinson’s Disease in the Older Patient.

[6] Fahn S, Elton R (1987). Members of the UPDRS development committee unified Parkinson’s rating scale. Recent Dev. Parkinson’s Dis..

[7] Rizzo G (2016). Accuracy of clinical diagnosis of parkinson disease: a systematic review and meta-analysis. Neurology.

[8] Kalia LV, Lang AE (2015). Parkinson’s disease. Lancet.

[9] Schrag A, Horsfall L, Walters K, Noyce A, Petersen I (2015). Prediagnostic presentations of parkinson’s disease in primary care: a case–control study. Lancet Neurol..

[10] Fox SH, Lang AE (2014). Dont delay, start today: delaying levodopa does not delay motor complications. Brain.

[11] Farzanehfar P (2018). Objective measurement in routine care of people with parkinson’s disease improves outcomes. NPJ Parkinson’s Dis..

[12] Bot BM (2016). The mpower study, Parkinson disease mobile data collected using researchkit. Sci. Data.

[13] Monahan T, Fisher JA (2010). Benefits of observer effects: lessons from the field. Qual. Res..

[14] Zhan, A. *et al.* High frequency remote monitoring of Parkinson’s disease via smartphone: Platform overview and medication response detection. arXiv preprint arXiv:1601.00960 (2016).

[15] Mazzoni P, Shabbott B, Cortés JC (2012). Motor control abnormalities in Parkinson’s disease. Cold Spring Harb. Perspect. Med..

[16] Maetzler W, Hausdorff JM (2012). Motor signs in the prodromal phase of Parkinson’s disease. Mov. Disord..

[17] Arroyo-Gallego T (2018). Detecting motor impairment in early parkinson’s disease via natural typing interaction with keyboards: validation of the neuroqwerty approach in an uncontrolled at-home setting. J. Med. Internet Res..

[18] Iakovakis D (2018). Touchscreen typing-pattern analysis for detecting fine motor skills decline in early-stage Parkinson’s disease. Sci. Rep..

[19] Iakovakis D (2018). Motor impairment estimates via touchscreen typing dynamics toward Parkinson’s disease detection from data harvested in-the-wild. Front. ICT.

[20] LeCun Y, Bengio Y, Hinton G (2015). Deep learning. Nature.

[21] Kiryu S (2019). Deep learning to differentiate parkinsonian disorders separately using single midsagittal mr imaging: a proof of concept study. Eur. Radiol..

[22] Papadopoulos, A. *et al.* Detecting parkinsonian tremor from IMU data collected in-the-wild using deep multiple-instance learning. *IEEE J. Biomed. Health Inf.* (2019). 10.1109/JBHI.2019.2961748 31880570

[23] Johri, A., Tripathi, A. *et al.* Parkinson disease detection using deep neural networks. In *2019 Twelfth International Conference on Contemporary Computing (IC3)*, 1–4 (IEEE, 2019).

[24] Iakovakis, D. *et al.* Keystroke timing and pressure data captured during touchscreen typing by early Parkinson’s disease patients and healthy controls. 10.5281/zenodo.2571623 (2019).

[25] Tassi P, Muzet A (2000). Sleep inertia. Sleep Med. Rev..

[26] Giancardo L, Sánchez-Ferro A, Butterworth I, Mendoza C, Hooker JM (2015). Psychomotor impairment detection via finger interactions with a computer keyboard during natural typing. Sci. Rep..

[27] Sabbe B, Hulstijn W, Van Hoof J, Zitman F (1996). Fine motor retardation and depression. J. Psychiatr. Res..

[28] Feldmann R, Weglage J, Roth J, Foell D, Frosch M (2005). Systemic juvenile rheumatoid arthritis: cognitive function and social adjustment. Ann. Neurol..

[29] Contreras-Vidal JL, Teulings H, Stelmach G (1998). Elderly subjects are impaired in spatial coordination in fine motor control. Acta Psychol..

[30] Ketcham, C. J. & Stelmach, G. E. Movement control in the older adult. In *Technology for adaptive aging* (National Academies Press (US), 2004).

[31] Seidel SE (2012). Subject-investigator reproducibility of the unified Parkinson’s disease rating scale. Parkinson. Relat. Disord..

[32] Goetz CG (2008). Movement disorder society-sponsored revision of the unified parkinson’s disease rating scale (mds-updrs): scale presentation and clinimetric testing results. Mov. Disord..

[33] Simonyan, K., Vedaldi, A. & Zisserman, A. Deep inside convolutional networks: Visualising image classification models and saliency maps. arXiv preprint arXiv:1312.6034 (2013).

[34] Pérez-López C (2016). Assessing motor fluctuations in parkinson’s disease patients based on a single inertial sensor. Sensors.

[35] Titova N, Chaudhuri KR (2017). Personalized medicine in parkinson’s disease: time to be precise. Mov. Disord..

[36] Matarazzo M (2019). Remote monitoring of treatment response in parkinson’s disease: the habit of typing on a computer. Mov. Disord..

[37] Kreiss D, Anderson L, Walters J (1996). Apomorphine and dopamine d1 receptor agonists increase the firing rates of subthalamic nucleus neurons. Neuroscience.

[38] Gottwald MD, Aminoff MJ (2011). Therapies for dopaminergic-induced dyskinesias in Parkinson disease. Ann. Neurol..

[39] Reichmann H (2011). View point: etiology in Parkinson’s disease dual hit or spreading intoxication. J. Neurol. Sci..

[40] Holmes CS, Hayford JT, Gonzalez JL, Weydert JA (1983). A survey of cognitive functioning at different glucose levels in diabetic persons. Diabetes Care.

[41] de Paula JJ (2016). Impairment of fine motor dexterity in mild cognitive impairment and Alzheimer’s disease dementia: association with activities of daily living. Braz. J. Psychiatry.

[42] Sperling R, Mormino E, Johnson K (2014). The evolution of preclinical alzheimer’s disease: implications for prevention trials. Neuron.

[43] Dorsey ER, Papapetropoulos S, Xiong M, Kieburtz K (2017). The first frontier: digital biomarkers for neurodegenerative disorders. Digital Biomark..

[44] Beauchet, O. *et al.* Motor phenotype of decline in cognitive performance among community-dwellers without dementia: population-based study and meta-analysis. *PLoS One* **9** (2014). 10.1371/journal.pone.0099318 PMC4049832 24911155

[45] Mastoras R-E (2019). Touchscreen typing pattern analysis for remote detection of the depressive tendency. Sci. Rep..

[46] Hill LJ (2016). The relationship between manual coordination and mental health. Eur. Child Adolesc. Psychiatry.

[47] Voigt, P. & Von dem Bussche, A. The EU general data protection regulation (gdpr). A Practical Guide, 1st Ed. (Springer International Publishing, Cham, 2017).

[48] Baldi, P. Autoencoders, unsupervised learning, and deep architectures. *Proceedings of ICML workshop on unsupervised and transfer learning* 37–49 (2012).

[49] Bengio, Y. Rmsprop and equilibrated adaptive learning rates for nonconvex optimization. arXiv:1502.04390 (2015).

[50] Oliphant TE (2007). Python for scientific computing. Comput. Sci. Eng..

