# Supplementary material for: Screening of Parkinsonian subtle fine-motor impairment from touchscreen typing via deep learning
Source: Sci Rep. 2020 Jul 28;10:12623. doi: 10.1038/s41598-020-69369-1 (PMC7387517; doi:10.1038/s41598-020-69369-1)
Supplement: Supplementary file 1 — Supplementary Information. [file 41598_2020_69369_MOESM1_ESM.pdf]

Screening of Parkinsonian subtle fine-motor impairment from touchscreen typing via deep learning

Dimitrios Iakovakis[1], K. Ray Chaudhuri[2], Lisa Klingelhoefer[3], Sevasti Bostantzopoulou[4], Zoe Katsarou[5], Dhaval Trivedi[2], Heinz Reichmann[3], Stelios Hadjidimitriou[1], VasileiosCharisis[1], and Leontios J. Hadjileontiadis[6,1]

[1] Department of Electrical and Computer Engineering, Aristotle University of Thessaloniki, Thessaloniki, Greece.

[2] King's College London, Institute of Psychiatry, Psychology & Neuroscience and Parkinson Foundation Centre of Excellence, King's College Hospital, London, United Kingdom.

[3] Department of Neurology, Technical University Dresden, Dresden, Germany

[4] Third Neurological Clinic, G. Papanikolaou Hospital, Thessaloniki, Greece.

[5] Department of Neurology, Hippokration Hospital, Thessaloniki, Greece.

[6] Department of Electrical and Computer Engineering, Khalifa University of Science and Technology, Abu Dhabi, UAE.

Fine-motor impairment (FMI) is progressively expressed in early Parkinson's Disease (PD) patients and is now known to be evident in the immediate prodromal stage of the condition. The clinical techniques for detecting FMI may not be robust enough and here, we show that the subtle FMI of early PD patients can be effectively estimated from the analysis of natural smartphone touchscreen typing via deep learning networks, trained in stages of initialization and fine-tuning. In a validation dataset of 36,000 typing sessions from 39 subjects (17 healthy/22 PD patients with medically validated UPDRS Part III single-item scores), the proposed approach achieved values of area under the receiver operating characteristic curve (AUC) of 0.89 (95% confidence interval: 0.80-0.96) with sensitivity/specificity:0.90/0.83. The derived estimations result in statistically significant ($p<0.05$) correlation of 0.66/0.73/0.58 with the clinical standard UPDRS Part III items 22/23/31, respectively. Further validation analysis on 9 de novo PD patients vs.17 healthy controls classification resulted in AUC of 0.97 (0.93-1.00) with 0.93/0.90. For 253 remote study participants, with self-reported health status providing 252.000 typing sessions via a touchscreen typing data acquisition mobile app (iPrognosis), the proposed approach predicted 0.79 AUC (0.66-0.91) with 0.76/0.71. Remote and unobtrusive screening of subtle FMI via natural smartphone usage, may assist in consolidating early and accurate diagnosis of PD.

	De-Novo PD patients	Healthy	
n (total n = 26)	9	17	
Demographic characteristics			p-value
Women # (%)	3	7	$p = 0.97$
Men # (%)	6	10	$p = 0.97$
Avg. Age, years (std)	56 (8)	55 (9)	$p = 0.3$
Subject #/# who completed education level H/U	2/7	4/13	$p = 0.7$
Subject #/#/#/#/# with smartphone experience level E1/E2/E3	1/1/7	0/0/13	$p = 0.13$
Avg. sum of UPDRS Part III (std)	12 (5)	1.3 (2.1)	$p < 0.05$
dBSi mean (std)/<br>dAFSi mean (std)/<br>dRSi mean (std)	0.52(0.41)/<br>1.01(0.30)/<br>0.92(0.43)	0.17(0.04)/<br>0.30(0.19)/<br>0.31(0.12)	$p < 0.001$

Table S1: Demographics table of TS_2

	PD patients	Healthy
n (total n = 253)	67	186
Demographic characteristics		
Women # (%)	24	67
Men # (%)	43	117
Avg. Age, years (std)	61 (7)	58 (7.5)
Subject #/# who completed education level H/U	25/42	53/133
Subject #/#/#/#/# with smartphone experience level E1/E2/E3	4/4/59	6/3/177
Data contribution		
Avg. valid recordings (std)	231	405

Table S2: Demographics table of TS_3

```
                           Logit Regression Results
==============================================================================
Dep. Variable:                  Label   No. Observations:                   39
Model:                          Logit   Df Residuals:                       34
Method:                           MLE   Df Model:                            4
Pseudo R-squ.:                    inf   Log-Likelihood:                -5484.7
converged:                      False   LL-Null:                        0.0000
Covariance Type:            nonrobust   LLR p-value:                     1.000
==============================================================================
        coef    std err          z      P>|z|      [0.025      0.975]
------------------------------------------------------------------------------
const         46.2474   7.59e+05   6.09e-05      1.000   -1.49e+06    1.49e+06
Age            0.0223      0.053      0.419      0.675      -0.082       0.126
Use          -25.1029    3.8e+05  -6.61e-05      1.000   -7.44e+05    7.44e+05
Gender         0.1514      1.071      0.141      0.888      -1.947       2.250
dRSi           4.1826      1.455      2.874      0.004       1.330           7

                           Logit Regression Results
==============================================================================
Dep. Variable:                  Label   No. Observations:                   39
Model:                          Logit   Df Residuals:                       34
Method:                           MLE   Df Model:                            4
Pseudo R-squ.:                    inf   Log-Likelihood:                -5366.1
converged:                      False   LL-Null:                        0.0000
Covariance Type:            nonrobust   LLR p-value:                     1.000
==============================================================================
        coef    std err          z      P>|z|      [0.025      0.975]
------------------------------------------------------------------------------
const         43.4143   4.19e+05      0.000      1.000   -8.22e+05    8.22e+05
Age            0.0376      0.053      0.713      0.476      -0.066       0.141
Use          -24.0509    2.1e+05     -0.000      1.000   -4.11e+05    4.11e+05
Gender        -0.0422      1.058     -0.040      0.968      -2.115       2.031
dAFSi          4.5383      1.623      2.796      0.005       1.357           7
                           Logit Regression Results
==============================================================================
Dep. Variable:                  Label   No. Observations:                   39
Model:                          Logit   Df Residuals:                       34
Method:                           MLE   Df Model:                            4
Pseudo R-squ.:                    inf   Log-Likelihood:                -4033.0
converged:                      False   LL-Null:                        0.0000
Covariance Type:            nonrobust   LLR p-value:                     1.000
==============================================================================
        coef    std err          z      P>|z|      [0.025      0.975]
------------------------------------------------------------------------------
const         47.8974   9.15e+05   5.24e-05      1.000   -1.79e+06    1.79e+06
Age            0.0235      0.053      0.447      0.655      -0.080       0.127
Use          -26.4710   4.57e+05  -5.79e-05      1.000   -8.97e+05    8.97e+05
Gender         0.4695      0.982      0.478      0.633      -1.455       2.394
dBSi          14.6698      7.121      2.060      0.039       0.714       28.62
```

Figure S1 Logistic regression tests using the subject status (PD or control) as dependent variable with the TS1 and Smartphone Usage (Use), age (Age) gender (Gender) and the prediction (dAF/R/B-Si) as independent variables.

Logit Regression Results

Dep. Variable:	Label	No. Observations:	26
Model:	Logit	Df Residuals:	21
Method:	MLE	Df Model:	4
Date:	Thu, 28 Nov 2019	Pseudo R-squ.:	inf
Time:	16:07:26	Log-Likelihood:	-inf
converged:	False	LL-Null:	0.0000
Covariance Type:	nonrobust	LLR p-value:	1.000

	coef	std err	z	P>|z|	[0.025	0.975]
const	49.7841	2.78e+05	0.000	1.000	-5.46e+05	5.46e+05
Age	-0.0788	0.105	-0.749	0.454	-0.285	0.127
Use	-26.1749	1.39e+05	-0.000	1.000	-2.73e+05	2.73e+05
Gender	-2.4017	4.352	-0.552	0.581	-10.931	6.127
**dRSi**	**15.8839**	**6.859**	**2.316**	**0.021**	**2.441**	**29.3**

Logit Regression Results

Dep. Variable:	Label	No. Observations:	26
Model:	Logit	Df Residuals:	21
Method:	MLE	Df Model:	4
Date:	Thu, 28 Nov 2019	Pseudo R-squ.:	inf
Time:	16:09:15	Log-Likelihood:	-inf
converged:	False	LL-Null:	0.0000
Covariance Type:	nonrobust	LLR p-value:	1.000

	coef	std err	z	P>|z|	[0.025	0.975]
const	34.2961	1.05e+05	0.000	1.000	-2.07e+05	2.07e+05
Age	-0.0669	0.113	-0.594	0.553	-0.288	0.154
Use	-19.0832	5.27e+04	-0.000	1.000	-1.03e+05	1.03e+05
Gender	-0.0886	1.504	-0.059	0.953	-3.036	2.859
**dBSi**	**45.7413**	**25.624**	**1.785**	**0.074**	**-4.481**	**95.964**

Logit Regression Results

Dep. Variable:	Label	No. Observations:	26
Model:	Logit	Df Residuals:	21
Method:	MLE	Df Model:	4
Date:	Thu, 28 Nov 2019	Pseudo R-squ.:	inf
Time:	16:10:14	Log-Likelihood:	-inf
converged:	False	LL-Null:	0.0000
Covariance Type:	nonrobust	LLR p-value:	1.000

	coef	std err	z	P>|z|	[0.025	0.975]
const	57.9487	1.74e+06	3.34e-05	1.000	-3.4e+06	3.4e+06
Age	-0.0791	0.137	-0.579	0.563	-0.347	0.189
Use	-30.8468	8.68e+05	-3.55e-05	1.000	-1.7e+06	1.7e+06
Gender	-2.8425	8.297	-0.343	0.732	-19.105	13.420
**dAFSi**	**13.2140**	**6.467**	**2.043**	**0.041**	**0.538**	**25.8**

Figure S2 Logistic regression tests using the subject status (PD or control) as dependent variable with the TS2 and Smartphone Usage (Use), age (Age) gender (Gender) and the prediction (dAF/R/B-Si) as independent variables.

Logit Regression Results

Dep. Variable:	Label	No. Observations:	253
Model:	Logit	Df Residuals:	248
Method:	MLE	Df Model:	4
Pseudo R-squ.:	inf	Log-Likelihood:	-inf
converged:	True	LL-Null:	0.0000
Covariance Type:	nonrobust	LLR p-value:	1.000

	coef	std err	z	P>|z|	[0.025	0.975]
const	-1.7256	1.317	-1.310	0.190	-4.307	0.856
Age	-0.0154	0.019	-0.795	0.426	-0.053	0.023
Use	-0.2342	0.354	-0.662	0.508	-0.927	0.459
Gender	0.1256	0.351	0.358	0.721	-0.563	0.814
**dRSi**	**2.5578**	**0.400**	**6.390**	**0.000**	**1.773**	**3.34**

Logit Regression Results

Dep. Variable:	Label	No. Observations:	253
Model:	Logit	Df Residuals:	248
Method:	MLE	Df Model:	4
Pseudo R-squ.:	inf	Log-Likelihood:	-64909.
converged:	True	LL-Null:	0.0000
Covariance Type:	nonrobust	LLR p-value:	1.000

	coef	std err	z	P>|z|	[0.025	0.975]
const	-1.2623	1.220	-1.035	0.301	-3.654	1.129
Age	-0.0004	0.018	-0.022	0.982	-0.035	0.035
Use	-0.2640	0.323	-0.818	0.413	-0.896	0.368
Gender	-0.0642	0.328	-0.195	0.845	-0.708	0.579
**dBSi**	**2.3348**	**0.471**	**4.956**	**0.000**	**1.411**	**3.25**

Logit Regression Results

Dep. Variable:	Label	No. Observations:	253
Model:	Logit	Df Residuals:	248
Method:	MLE	Df Model:	4
Pseudo R-squ.:	inf	Log-Likelihood:	-inf
converged:	True	LL-Null:	0.0000
Covariance Type:	nonrobust	LLR p-value:	1.000

	coef	std err	z	P>|z|	[0.025	0.975]
const	-1.8121	1.312	-1.381	0.167	-4.384	0.760
Age	-0.0130	0.019	-0.687	0.492	-0.050	0.024
Use	-0.2722	0.357	-0.762	0.446	-0.973	0.428
Gender	0.0099	0.346	0.029	0.977	-0.668	0.688
**dAFSi**	**2.9731**	**0.468**	**6.356**	**0.000**	**2.056**	**3.89**

Figure S3 Logistic regression tests using the subject status (PD or control) as dependent variable with the TS3 and Smartphone Usage (Use), age (Age) gender (Gender) and the prediction (dAF/R/B-Si) as independent variables.
